# Orbital MRI Findings in a Patient With Giant Cell Arteritis (GCA): A Case Report

**DOI:** 10.7759/cureus.49507

**Published:** 2023-11-27

**Authors:** Saleh Alhawiti, Toka AlSulaim

**Affiliations:** 1 Rheumatology Department, Prince Sultan Military Medical City, Armed Forces Health Services, Riyadh, SAU; 2 Rheumatology Department, King Abdulaziz Medical City Riyadh, Ministry of National Guard Health Affairs, Riyadh, SAU

**Keywords:** giant cell arteritis, headache, orbital mri, optic perineuritis, optic chiasm

## Abstract

Giant cell arteritis (GCA) is a medium- and large-vessel systemic vasculitis. It is common among people aged 50 years and older. GCA presents with cranial manifestations of headache, visual symptoms, cerebrovascular events, and systemic manifestations. The diagnosis of GCA is confirmed with a positive temporal artery biopsy. Treatment of GCA consists of high-dose steroids with slow tapering over 18 to 24 months and steroid-sparing therapy to maintain remission. Due to the risk of the most feared complication of GCA, irreversible visual loss, once GCA is suspected, high-dose steroids should be commenced immediately, and a temporal artery biopsy should be done not beyond one to two weeks after starting steroids to avoid the effects of steroids on the result of the temporal artery biopsy. There are orbital radiological findings reported in patients with an atypical presentation of GCA on MRI; these findings include non-specific orbital inflammation, optic nerve inflammation, optic nerve sheath inflammation, and optic chiasm enhancement.

## Introduction

Giant cell arteritis (GCA) is a medium- and large-vessel systemic vasculitis primarily affecting people aged 50 years or older. It is commonly documented in Scandinavian countries and among people of Scandinavian descent. Also, it is more common among people of northern European origin. GCA is the most common vasculitis in people aged 50 years and older. Females tend to have more GCA than males, with a reported female-to-male ratio ranging from 1.4:1 to 2.9:1 [1-4]. The average annual incidence reported in a study conducted in Sweden among 665 patients with biopsy-proven GCA between 1976 and 1995 was 22.2/100,000 (female 29.8 and male 12.5). In another study conducted in Spain among 255 patients with biopsy-proven GCA between 1981 and 2005, they reported a lower incidence of GCA (10.1) and nearly equal incidence between females and males, 10.2 and 9.9, respectively [5,6]. The typical presentation of GCA is temporal arteritis, presenting with a broad spectrum of clinical and laboratory abnormalities attributable to ischemia and systemic inflammation [7]. Various presentations of GCA have been described, including an (occult) variant with mainly ocular manifestations in the absence of systemic symptoms and signs and a (silent) variant characterized by pronounced systemic manifestations and the absence of typical cranial manifestations [7-10]. Many studies reported MRI findings of the intraorbital structures in patients with biopsy-proven GCA. These reported findings include inflammation of the optic nerve, optic nerve sheath, optic chiasm, and/or intraorbital fat [11].

This case report describes the clinical presentation and orbital MRI findings of a patient with a biopsy-proven GCA.

## Case presentation

A 75-year-old female presented to the emergency department with a recent history of a new-onset headache and fever for three weeks. More details revealed a constant holocephalic pressure-like headache that increased with movement and changing position and partially responded to acetaminophen. This headache is associated with bilateral blurry vision, bilateral eye pain, and a documented fever of 38°C. The fever is associated with night sweats, poor appetite, fatigability, and significant weight loss of about 12 kg over a short period of time. Recently, she was diagnosed with left eye glaucoma when she complained of redness, pain, and blurring of vision and was prescribed anti-glaucoma treatment. She has no jaw or limb claudication, weakness or stiffness, arthralgia or skin rash, and facial symptoms or diplopia. The patient has no respiratory, gastrointestinal, or genitourinary symptoms. She has a background of bilateral carpal tunnel syndrome, dyslipidemia, and knee osteoarthritis with an old right total knee replacement. An urgent lumbar puncture was done in the emergency to rule out meningitis, and the results were unremarkable. The patient was admitted to the hospital with a fever of unknown origin for investigation. Clinical examination showed normal hemodynamics and a low-grade fever. There was no temporal artery tenderness, and temporal arteries were palpable bilaterally. Mild redness was observed in her right eye. The ophthalmological examination showed visual acuity: OD 20/50, OS 20/60, with mild optic disc swelling, macular edema, and vascular tortuosity.

In summary, our patient is a 75-year-old female with a subacute presentation of non-specific headache, bilateral eye pain with blurry vision, and constitutional symptoms preceded by a diagnosis of glaucoma. The examination was remarkable for low-grade fever, right eye redness, mild optic disc swelling, macular edema, and vascular tortuosity.

Investigations

Laboratory results (Table 1) were significant for increased white blood cells, mainly neutrophils and eosinophils. Platelets were normal and hemoglobin was low. Inflammatory markers were very high, including erythrocyte sedimentation rate, C-reactive protein, and procalcitonin. Extensive workups for underlying infectious causes were unremarkable, including blood cultures, urine cultures, sputum cultures, mycobacterium tuberculosis QuantiFERON (TB QuantiFERON), malaria antigens, brucella serology, hepatitis B virus (HBV), hepatitis C virus (HCV), cytomegalovirus (CMV), Epstein-Barr virus (EBV), COVID-19, and Middle East respiratory syndrome coronavirus (MERS-COV). The lumbar puncture was unremarkable. Serological workup came negative for antinuclear antibody, anti-double strand DNA, anti-smith, anti-Ro/SSA, anti-La/SSB, rheumatoid factor, anti-cyclic citrullinated peptide, and anti-neutrophil cytoplasmic antibodies (MPO-ANCA and PR3-ANCA). The IgG level was mildly increased, and the IgE was markedly increased.

**Table 1:** Laboratory results WBC: white blood cells, ESR: erythrocyte sedimentation rate, CRP: C-reactive protein, TB QuantiFERON: mycobacterium tuberculosis QuantiFERON, HBVsAg: hepatitis B virus surface antigen, HBVsAg antibody: hepatitis B virus surface antigen antibody, HCV antibody: hepatitis C virus antibody, CMV IgM: cytomegalovirus immunoglobulin M, CMV IgG: cytomegalovirus immunoglobulin G, EBV PRC: Epstein-Barr virus polymerase chain reaction, COVID-19: coronavirus disease 19, MERS-COV: Middle East respiratory syndrome coronavirus, ANA: antinuclear antibody, anti-dsDNA: anti-double stranded deoxyribonucleic acid antibody, anti-SM: anti-smith, anti-Ro/SSA: anti Sjögren syndrome antibody A, anti-La/SSB: anti-Sjögren syndrome antibody B, RF: rheumatoid factor, anti-CCP: anti-cyclic citrullinated peptide, MPO-ANCA: anti-neutrophil cytoplasmic antibody (myeloperoxidase), PR3-ANCA: anti-neutrophil cytoplasmic antibody (proteinase 3), IgG: immunoglobulin G, IgE: immunoglobulin E

Test	Result	Reference range
Hematology		
WBC	13.1	4.5-11 × 10^9^/L
Neutrophils	8.5 (65%)	2.5-8 × 10^9^/L (55-70%)
Eosinophils	1.37 (10.4%)	0.05-0.5 × 10^9^/L (1-4%)
Hemoglobin	111	120-160 gm/L
Platelets	315	150-400 × 10^9^/L
ESR	120	<20 mm/h
CRP	141	<8 mg/L
Procalcitonin	0.21	<0.05 ng/ml
Infectious workup		
Cultures (blood, urine, and sputum)	No growth	No growth
TB QuantiFERON	Negative	Negative
Brucella serology	Negative	Negative
Malaria antigen detection test	Negative	Negative
HBVsAg and HBVsAg antibody	Negative	Negative
HCV antibody	Negative	Negative
CMV IgM	Not detected	Not detected
CMV IgG	>180	Undetectable
EBV PCR	Not detected	Not detected
COVID-19	Negative	Negative
MERS-COV	Negative	Negative
Serology		
ANA	Negative	Negative
Anti-dsDNA	Negative	Negative
Anti-SM	Negative	Negative
anti-Ro/SSA and anti-La/SSB	Negative	Negative
RF	Negative	Negative
Anti-CCP	Negative	Negative
MPO-ANCA	Negative	Negative
PR3-ANCA	Negative	Negative
IgG level	17	6-16 g/L
IgE level	1315	<300 UI/ml

**Figure 1:**
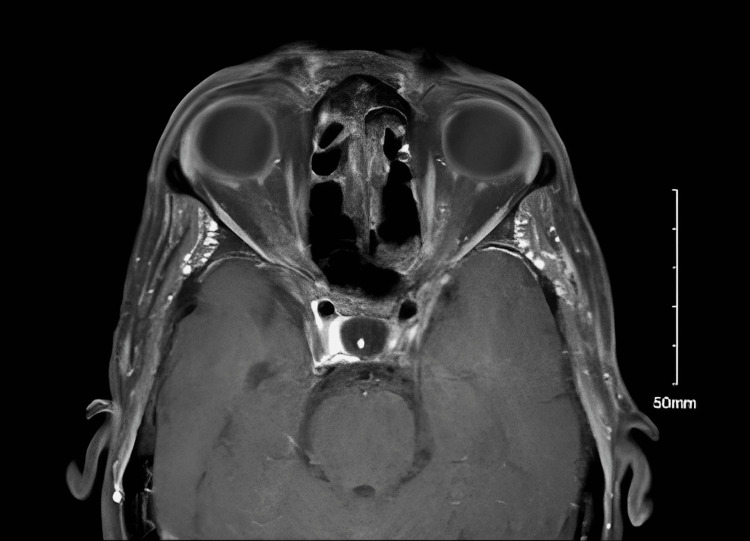
Orbital MRI with contrast, cross-sectional view This slide shows left optic nerve peripheral enhancement with adjacent fat stranding. Similar findings were found in the right optic nerve in the other slides (pictures not shown)

The brain CT was normal, and the sinus CT showed a thickening of the paranasal sinuses. After one week of extensive investigation and an empirical course of broad-spectrum antibiotics, there was no change in her clinical status. An orbital MRI was done, and a right temporal artery biopsy was performed. Interestingly, orbital MRI revealed bilateral optic perineuritis, with bilateral optic nerve peripheral enhancement and adjacent fat strandings extending to the optic chiasm (Figure 1).

There was a questionable enhancement of optic chiasm that was not clear in all sections of the MRI, which could be an artifact versus a true enhancement after discussion with the neuroimaging physician. Additionally, there were bilateral small cystic lesions on the lateral aspect of the globes at the insertion of the lateral rectus muscles. The patient started on methylprednisolone 80 mg immediately, then maintained on methylprednisolone 80 mg every eight hours, followed by prednisolone 60 mg daily. Later, the result of her temporal artery biopsy came back positive for GCA. The histopathology of the temporal artery biopsy showed a muscular artery with a narrowed lumen, intimal thickening, disruption of internal elastic lamina, lymphoplasma histiocytic aggregates, and dystrophic calcifications. The patient was started on tocilizumab 162 mg subcutaneous injection every week and continued her high-dose oral steroids. The follow-up assessment showed a dramatic response to all of her symptoms, and the last ophthalmology examination revealed a clear fundus examination with a healthy optic disc and macula bilaterally.

## Discussion

GCA is the most common vasculitis in people aged 50 years or older. The most feared complication of GCA is irreversible visual loss, along with other vascular complications such as cerebrovascular events and arterial aneurysms that may rupture. Many case reports and a recent case series reported different orbital MRI findings in patients with biopsy-proven GCA. The first case of optic perineuritis was reported in 2002 in an 83-year-old male who developed neck pain, followed by acute bilateral visual loss and a fever of 38.3°C. Despite the treatment with intravenous steroids, the patient's visual loss progressed, and MRI orbits revealed bilateral optic nerve sheath enhancement. An optic nerve sheath biopsy was performed and showed the presence of giant cells. Because of the presence of these giant cells in the optic sheath, bilateral temporal artery biopsies were done for him, and the results came back positive for GCA [12].

Another case report of a patient with mainly visual symptoms and a unilateral headache underwent an orbital MRI for evaluation. The results of the orbital MRI revealed bilateral optic perineuritis. Bilateral temporal artery biopsies were done for him to look for possible GCA, and the results of both biopsies came back negative for GCA. Consequently, this patient was labeled as having bilateral idiopathic optic perineuritis based on his clinical presentation, neuroimaging, negative temporal artery biopsy bilaterally, and the absence of a causative infectious cause. As a treatment for the proposed diagnosis (bilateral idiopathic optic perineuritis), the patient was treated with high-dose intravenous pulse steroids followed by prednisolone 1 mg/kg/day. Later, shortly after the treatment commenced, prednisolone was interrupted due to bacterial meningitis. The patient completed a course of antibiotics for the active infection and was then discharged home. A few weeks after discharge, the patient developed classical symptoms of GCA. He presented with a left severe temporal headache and jaw claudication, and examination showed bilateral mild skin necrosis over the temporal regions. Oral prednisolone resumed and led to significant improvement of his new symptoms (left temporal headache and jaw claudication). This patient was diagnosed with GCA based on his clinical presentation and the dramatic response to steroids [13].

In a recent case series investigating the intraorbital MRI findings in patients with biopsy-proven GCA, the study reported four main orbital MRI findings in GCA as follows: non-specific orbital inflammation, optic nerve enhancement, optic nerve sheath enhancement, and the first reported case of optic chiasmal enhancement [14]. These intraorbital MRI findings can be seen in infiltrative, inflammatory, demyelinating, infectious, and neoplastic diseases.

## Conclusions

GCA carries high morbidity and mortality if untreated or even if treatment is delayed. The most-feared complication is irreversible visual loss. Early diagnosis and treatment help to control the disease and prevent complications, including saving sight of patients. In the context of GCA and its variable and sometimes atypical presentations, intraorbital MRI findings may help guide clinicians to suspect GCA and avoid delay in the diagnosis and treatment. In this case, we present the intraorbital findings of a patient with biopsy-proven GCA who has bilateral perineuritis and intraorbital findings. The chiasmal enhancement was not clear in all sections, and it was questionable and not confirmed whether it was a true enhancement or an artifact.
